# Biomarkers for detecting prostate cancer: Protocol for an umbrella review with integrated network meta-analysis

**DOI:** 10.1097/MD.0000000000016517

**Published:** 2019-07-26

**Authors:** Junhai Jia, Yue Sun, Jingjie Ren, Muyang Li, Jiancheng Wang, Haiyang Li

**Affiliations:** a Gansu Province Hospital Rehabilitation Center; b School of Nursing, Lanzhou University; c The Second Clinical Medical College of Lanzhou University; d Hospital Management Research Center, Lanzhou University; e Gansu Provincial Hospital, Lanzhou, Gansu Province, China.

**Keywords:** biomarker, diagnostic test accuracy, prostate cancer, systematic reviews

## Abstract

**Background:**

Prostate cancer (PCa) is common, with it being the 2nd most prevalent cancer in men worldwide and the 6th leading cause of death in men. Screening for any type of cancer aims to increase the chances of successful treatment through early detection of the disease. There were some systematic reviews (SRs) evaluated the diagnostic value of biomarkers for the diagnosis of PCa and no studies have been conducted to analyze the quality of these SRs. We are not clear which kind of marker is the best choice. Thus, this study aims to assess the methodologic quality of the SRs and reanalyze the published data based on SRs for the biomarkers to find the optimal biomarker for the early diagnosis of PCa.

**Methods:**

We performed a systematic literature search of PubMed, Embase, Web of Science, and Cochrane Library and to identify relevant SRs from inception to April 2019. Diagnostic accuracy studies included any type of single biomarker or combined biomarkers aimed at evaluating the diagnostic value is considered eligible for this overview. The Assessment of Multiple Systematic Reviews-2 (AMSTAR-2) instrument will be used to evaluate the risk of bias of the included SRs. Standard pairwise meta-analysis and adjusted indirect comparison will be used to compare the diagnostic value of different biomarkers.

**Results:**

The results of this study will be submitted to a peer-reviewed journal for publication.

**Conclusion:**

This study will reanalyze the published data based on SRs. We hope that the results will help find a biomarker with the superior diagnostic performance for the diagnosis of PCa.

**PROSPERO registration number:**

CRD42019125880.

## Abstract 2

Supplemental Digital Content is available in the text

## 1 Introduction

Prostate cancer (PCa) refers to an epithelial malignancy that occurs in the prostate. Pathologic types of PCa include adenocarcinoma (alveolar adenocarcinoma), ductal adenocarcinoma, urothelial carcinoma, squamous cell carcinoma, and adenosquamous carcinoma. Adenocarcinoma of the prostate is common, with it being the 2nd most prevalent cancer in men worldwide and the 6th leading cause of death in men.^[1–3]^ The high mortality rate of patients with PCa is due primarily to the fact that the disease usually becomes clinically apparent after it has metastasized. The 5-year relative survival rates of patients with localized and regional PCa can reach 100%, but among metastatic PCa patients, the 5-year survival rate is much lower at 30%.^[4]^ Screening for any type of cancer aims to increase the chances of successful treatment through early detection of the disease.^[5–7]^ The use of the biomarker and of advanced imaging techniques such as multiparametric and whole-body magnetic resonance imaging for the detection of PCa is a research hotspot in recent years.^[8]^

Prostate-specific antigen-based PCa screening remains a controversial topic. Up to now, there is worldwide consensus on the statement that the harms of population-based screening, mainly as a result of overdiagnosis (the detection of clinically insignificant tumors that would have never caused any symptoms), outweigh the benefits.^[9]^ A number of biomarkers are currently available for PCa diagnosis, and the most common of which include using prostate-specific antigen, cell-free DNA, and microRNAs (miRNAs).^[10–14]^ Apart from the unbearable physiologic and psychologic inconvenience caused by PCa screening, the increased financial costs for health care systems globally should be taken into account as well. Thus, the right choice of new cost-efficient and accurate diagnostic approaches for PCa is urgently needed.^[15]^

Network meta-analysis has been considered to extend conventional meta-analysis on multiple treatments (i.e., 3 or more) for a given condition.^[16–19]^ The current “umbrella” reviews aim to synthesize the findings from multiple reviews and provide clinicians a report which summarizes the states of knowledge.^[20]^ There were some systematic reviews (SRs) evaluated the diagnostic value of biomarkers for the diagnosis of PCa and no studies have been conducted to analyze the quality of these SRs.^[21–24]^ We are not clear which kind of marker is the best choice. Thus, this study aims to assess the methodologic quality of the SRs and reanalyze the published data based on SRs for the biomarkers to find the optimal biomarker for the early diagnosis of PCa.

## 2 Methods

We will reanalyze and compare the published data of SRs of diagnostic accuracy of the different hormonal biomarker for PCa. This research protocol will fully follow the Preferred Reporting Items for Systematic Reviews and Meta-analysis Protocols (PRISMA-P) checklist.^[25]^ The protocol for this meta-analysis was registered on PROSPERO (International Prospective Register of Systematic Reviews) and the registration number is CRD42019125880. Ethics approval and patient consent are not required as this study is an overview based on published SRs.

### 2.1 Eligibility criteria for this review

#### 2.1.1 Type of studies

We will include SRs, which must include meta-analytical results and meet the participants, index tests, and outcomes of interest criteria described as follows. SRs that only report data narratively will be excluded.

#### 2.1.2 Participants

Study participants who diagnosed with PCa according to pathologic histology examination will be included. People with distant metastasis of PCa will be excluded. There are no limitations in age, race, nation, sex, and nationality of participates, as well as treatment plan and stage of cancer.

#### 2.1.3 Index tests

Any type of single biomarker or combined biomarkers aimed at evaluating the diagnostic value is considered eligible for this overview. However, 1 biomarker combined imaging patterns or other indicators will be excluded.

#### 2.1.4 Outcome measures

The primary outcomes were diagnostic value of sensitivity (SEN), specificity (SPE), diagnostic odds ratio (DOR) and their respective 95% confidence intervals (CIs) or true positive (TP), false positive (FP), true negative (TN), and false negative (FN) values which allow us to calculate the diagnostic performance indices for each include primary study.

#### 2.1.5 Exclusion criteria

The exclusion criteria were as follows: SRs without meta-analysis; SRs that did provide sufficient information to allow us to calculate the TP, FP, TN, and FN values; publications without complete data; protocols, review articles, conference abstracts, guidelines, consensus, documents or expert position papers, summaries, comments, letters, brief reports, and proceeding studies; and duplicated articles.

### 2.2 Search methods for identification of studies

The search strategies for relevant SRs were conducted by an information specialist librarian. A systematic search was performed using PubMed, Embase, Web of Science, and Cochrane Library to identify relevant SRs from inception to April 2019. There were no limitations on publication language and the year of publication. The references of relevant SRs/meta-analyses were searched to identify additional potential studies. Full details of the literature search strategies the PubMed were shown in Supplemental 1.

### 2.3 Selection of studies

We managed all retrieved titles and abstracts with the reference manager software EndNote (Version X7, Thomson Reuters). Two authors independently screened the titles and the abstracts. If a title or abstract appeared to meet the eligibility criteria for inclusion in the review, or we could not determine eligibility, a full-text version of the article was obtained and assessed by 2 authors (JH-J and YS) to determine whether it met the inclusion criteria. We resolved discordant evaluations by discussion to reach consensus.

### 2.4 Data extraction and management

A draft data extraction sheet will be developed using Microsoft Excel 2013 (Microsoft Corp, Redmond, WA, www.microsoft.com). Two reviewers will independently extract study characteristics from the included SRs including: author name, number of authors, publication year, journal name, country of the journal, funding, and types of included studies, number of included studies, and number of participants, baseline diagnosis (age, sex, and location), number and name of biomarkers, results of statistical analysis including sensitivity, specificity, likelihood ratio, predictive value, DOR, and area under curve. If we find that multiple reviews are identified for the same research question but share the same primary study, the repeated and identical data that overlaps the original study will only be included once. For the updated original study, the most recent study will be selected for data extraction, and the old version will be used as supplemental information if needed. If diagnostic performance indices in each original study were not found, we will use the number of TP, FP, TN, FN to calculate sensitivity, specificity, and DOR. For missing or unclear data, we will contact the research author for access. The difference will be resolved by consensus. If there remains any discrepancy, the 3rd auditor will make a consensus decision.

### 2.5 Assessment of methodologic quality

We will assess the methodologic quality of included SRs using Assessment of Multiple Systematic Reviews-2 (AMSTAR-2) instrument.^[25–27]^ This checklist contains 7 critical domains with 16 items. The overall confidence of the results of the review will be classified as high, moderate, low, and critically low. To indicate the degree of compliance, each checklist item will be assigned one of the following 3 responses: “Yes,” “No,” or “Partial Yes.” The quality assessment of the included SRs will be performed independently by 1 reviewer and verified by another, and the differences will be resolved through discussion to reach a consensus.

### 2.6 Statistical analysis and data synthesis

#### 2.6.1 Pairwise meta-analysis

Data of sensitivity, specificity, DOR, positive likelihood ratio, negative likelihood ratio, and their 95% CI lower limit, 95% CI upper from each SR will be used to perform the pairwise meta-analysis. We will generate the forest plots to present the diagnostic indices for each biomarker and present 95% CIs for all outcomes. The *I*^2^ test will be used to analyze heterogeneity between studies evaluated with the Chi-squared test. If the *I*^2^ is <50%, the effect size will be estimated using a fixed-effect model. If we find considerable heterogeneity among the studies, we will conduct subgroup analyses to explore the sources of heterogeneity. Random effects model, conduct sensitivity analysis, and subgroup analysis will be used to detect the source of heterogeneity. Otherwise, our review team will explore clinical heterogeneity. All analyses and plots will be generated using STATA (13.0; Stata Corporation, College Station, TX).

#### 2.6.2 Network meta-analysis

Relative sensitivity, relative specificity, and relative DOR between different biomarkers will be first calculated using STATA (13.0; Stata Corporation). Then, we will use the relative diagnostic indices to make the indirect comparison. If data were allowed, we will conduct a network meta-analysis.

#### 2.6.3 Subgroup analysis

We will identify subgroup analyses based on the primary studies reporting subgroup analysis results and extract data from these studies. If sufficient data extracted from the primary studies allow, we will conduct a subgroup analysis, including patient's gender, age, weight, country of study, treatment plan, and biomarker truncation and explore these will affect the diagnostic value of biomarkers.

### 2.7 Assessment of publication bias

If there are more than 10 SRs reported the diagnostic value of a biomarker, egger funnel plot method through Stata V.15.0 will be performed to help distinguish asymmetry due to publication bias.^[28]^

## 3 Result

**Figure 1:**
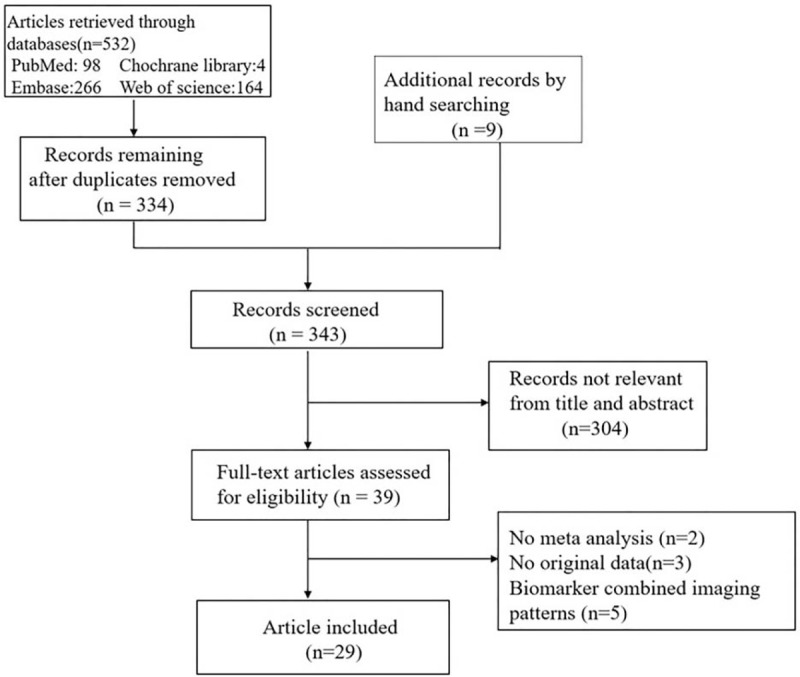
Summary of evidence search and selection.

Figure 1 shows the detailed results of the included SRs, where 29 SRs proved eligible.

## 4 Discussion

To reduce overdiagnosis and overtreatment of indolent PCa, while improving the detection of clinically significant PCa and reducing the number of biopsy procedures, we need more accurate diagnostic methods and better risk stratification.^[29]^ This document has outlined the methods for undertaking the overview and update of biomarkers for detecting PCa.

Ethics and dissemination: Ethics approval and patient consent are not required as this study is an overview based on published systematic reviews.

## Author contributions

**Data curation:** Junhai Jia, Yue Sun, Jingjie Ren.

**Formal analysis:** Junhai Jia.

**Investigation:** Haiyang Li.

**Methodology:** Yue Sun, Jiancheng Wang, Haiyang Li.

**Visualization:** Jingjie Ren, Muyang Li, Jiancheng Wang.

**Writing – original draft:** Junhai Jia, Yue Sun.

Haiyang Li orcid: 0000-0002-3974-2090.

## Supplementary Material

Supplemental Digital Content
